# A case–control study regarding factors associated with digital dermatitis in Norwegian dairy herds

**DOI:** 10.1186/s13028-022-00635-0

**Published:** 2022-08-13

**Authors:** Lina Ahlén, Ingrid Hunter Holmøy, Ane Nødtvedt, Åse Margrethe Sogstad, Terje Fjeldaas

**Affiliations:** 1 Department of Production Animal Clinical Sciences, Faculty of Veterinary Medicine, Norwegian University of Life Sciences, P.O. Box 5003, 1432 Ås, Norway; 2 Animalia, Økern, P.O. Box 396, 0513 Oslo, Norway

**Keywords:** Bovine digital dermatitis, Dairy cattle, Detection, Questionnaire, Risk factors

## Abstract

**Background:**

Digital dermatitis (DD) is a contagious bovine foot disease causing painful lesions, lameness, and reduced animal welfare. Previous studies indicate a complex aetiology of the disease. The aim of this study was to compare DD negative and DD positive herds to identify factors associated with DD in Norwegian dairy herds by analysing data obtained in a questionnaire and data recorded in the Norwegian Dairy Herd Recording System (NDHRS). The questionnaire was e-mailed to the owners of all 380 herds recorded with DD in 2019 and to 1530 randomly selected herds with no recordings of DD. Altogether 559 dairy herds were included in the final study population, of which 113 was classified as DD positive (≥ one cow recorded with DD in NDHRS) and 446 as DD negative. When stratified by housing system, the ratio between DD positive and negative herds was 108/281 in free stalls and 5/165 in tie stalls. Multilevel logistic regression was used to model the association between potential risk factors and variables related to the detection and diagnosis of DD, and the outcome in the free-stall population. Geographical area (county) was included in the model as a random effect.

**Results:**

In the final study population 108/113 (96%) of the DD positive herds were housed in free stalls versus 5/113 (4%) in tie stalls. The free-stall herds’ DD status was associated with purchase of cattle during the last 5 years (baseline: 0 animals, OR = 2.30 for category 12–27 animals, OR = 4.34 for 28–52 animals, and OR = 5.39 for  ≥ 53 animals). The DD status was also associated with claw trimming frequency (Baseline: 1 < 2/year, OR = 0.41 for category  < 1/year, and OR = 4.09 for  ≥ 3/year), whether the claw trimming was done by a certified professional trimmer or not (baseline:  ≤ 90% of the cows, OR = 3.98 for category  ≥ 90% of the cows), cleaning of feet in the chute before trimming (baseline: no cleaning, OR = 1.98 for category cleaning), and alley flooring (baseline: slatted floor, OR = 2.36 for category solid floor).

**Conclusions:**

Digital dermatitis was far more frequent in Norwegian dairy herds housed in free stalls versus those housed in tie stalls. In the free-stall herds purchase of cattle, increasing trimming frequency, use of certified professional trimmer, cleaning of the feet in the chute, and solid flooring in the alleys were associated with increased odds of recorded DD.

**Supplementary Information:**

The online version contains supplementary material available at 10.1186/s13028-022-00635-0.

## Background

Digital dermatitis (DD) is a contagious bovine foot disease causing ulcerative lesions in the heels, close to the coronary band or the skin in and around the interdigital cleft [1–3]. The lesions can be painful, resulting in lameness and reduced animal welfare, reduced milk and meat production and lowered fertility [4–8]. Previous studies indicate a complex aetiology of the disease involving DD-associated bacteria, hygiene and management routines, immunity of the host and genetic characteristics [9, 10]. The disease is endemic in almost every country with intensive milk production and has for the last 20 years also been an emerging problem in beef cattle [11, 12]. Studies from severely affected countries describe a situation with an endemic spread between farms and high numbers of affected animals per herd [13, 14]. Worst of all, when established in a herd, DD is difficult to eliminate and the efficacy of curative and preventive measures is uncertain [15].

External biosecurity is considered essential to control DD, and studies have shown that purchase of live animals is important for the dissemination and introduction of DD to naïve herds [16, 17]. A Danish study found that herds where the latest animal purchase occurred  ≤ 1 year ago were at increased odds of digital dermatitis compared to those in which the latest purchase happened  > 1 year ago [18]. Quarantine and claw examination before introducing new animals into herds are recommended [19]. The disease may also be transferred passively with poorly cleaned and disinfected hoof knives [20], and higher herd prevalence of DD has been found to be associated with animal transporters having access to the area where cattle are housed [18].

Important risk factors for within-herd spread are housing conditions and managements factors like hygiene, herd size, grazing- and trimming routines [9]. Relun et al. [5] found a direct relationship between levels of leg cleanliness in dairy cows (measured at herd level) and levels of DD, and disinfectant footbaths and washing of the feet has been documented to reduce the risk for DD [21–23].

The diagnostic method is important for correct recording of DD, and different procedures are introduced. The traditional method is inspection at trimming in chutes. However, the DD lesions have different stages and a dynamic appearance [24] and the diagnose is dependent on the trimmers’ diagnostic abilities, alertness and also whether the cows’ feet are washed or not [25]. Other methods are mirror scoring [25, 26] or use of a borescope [27] on cows standing in the parlour or pen. Good lighting is necessary to detect minor lesions [28].

An ongoing Norwegian DD study indicates a mild course of the disease with small lesions, low degree of lameness and relatively low prevalence in DD positive dairy herds [28]. There are major differences across Norway and a limited number of affected animals are recorded per herd. This might be explained by Norwegian herds having relative few animals, low milk production per animal and a substantial number of tie stalls compared to other countries [29]. However, the Norwegian dairy production is changing [30] from tie-stall to free-stall housing with larger herds and increased milk yield, making Norwegian dairy herds more susceptible for DD. Little is known about risk factors associated with DD in Norway and factors related to the detection of the disease. Consequently, more national knowledge is necessary to minimize the spread of the disease to naïve herds and prevent the development of more aggressive DD in Norway.

The aim of this study was to compare DD positive (DD+) and DD negative (DD−) herds to identify factors associated with DD in Norwegian dairy herds by analysing data obtained in a questionnaire and data recorded in the Norwegian Dairy Herd Recording System (NDHRS).

## Methods

### Study design and selection of herds

In this case–control study, the NDHRS was used to select cases and controls for identification of factors associated with DD, both risk factors and factors related to detection and diagnosis. In NDHRS, individual cows are diagnosed and recorded as DD− or DD+ by professional trimmers and veterinarians after trimming and examination of the feet. Their diagnoses are guided by the Nordic Claw Atlas [31], where DD is defined as “Dermatitis in, dorsal to or behind the interdigital cleft with erosions and bleeding, often painful lesions”. The four photos showed to illustrate DD in the atlas are all acute lesions (M2 or M1).

Individual claw trimming records from 2019 were aggregated to herd level to determine the DD status of each herd. A herd’s DD status became positive when one or more cows are recorded with DD during 2019. No exclusion criteria were set, meaning all members of NDHRS were eligible for inclusion in the study. All herds recorded with DD in 2019, a total of 380, were included in the study population as cases and 1530 DD negative herds were included as controls. Controls were selected from a population of 6870 dairy herds using computer-generated random numbers.

### Questionnaire design

The questionnaire consisted of 35 closed questions. The questions addressed issues like housing, management including secondary control measures, biosecurity, claw health, and trimming routines. If necessary, more than one answer could be given, and with room for comments where relevant. A pilot version was tested on five different dairy farmers and their feedback was used to improve the questions. The 4th of November 2019, the project partner TINE SA, e-mailed the questionnaire in Questback to all 1910 farmers included in the study population. The estimated time for completing the questionnaire was 15 min. The questionnaire was available in Questback until the 15th of December 2019 and during this period, reminders were e-mailed once a week. The complete questionnaire, translated into English, is provided in Additional file 1.

### Norwegian dairy herd recording system

Additional data was extracted from NDHRS by TINE SA. Herd level data retrieved from the recording system was geographical area (county in Norway), herd size, housing system, milking system, and average 305d milk yield in 2019. Records of claw trimming, infectious claw diseases (including DD), and use of artificial insemination (ai)/bull were retrieved at cow level. Individual animal records were aggregated to herd level, and included information on the frequency of claw trimming, the proportion of different infectious claw diseases (including heel horn erosion, interdigital dermatitis, interdigital hyperplasia, interdigital phlegmon and DD), and the proportion of mating performed by bull and ai, respectively. Furthermore, records of purchased animals in the period from Jan 2015 to Dec 2019 were extracted. The number of incoming animals in each age group (calf, heifer, young bull, bull, cow and unknown) and the total number of purchased animals were aggregated at herd level.

Data from Questback and NDHRS was separately imported to Stata for cleaning and quality control before being merged into one dataset for further processing and analyses. All data handling and statistical analyses were performed in Stata (Stata/SE 15.1, Stata Corp., College Station, TX).

### Descriptive statistics

Distribution by DD category (DD+ and DD−) stratified on housing system (free or tie stall) regarding housing conditions, management characteristics, trimming routines, and biosecurity is in Tables 1, 2, 3 and 4. Secondary control measures and other infectious claw diseases in the free-stall herds are in Tables 5 and 6, respectively.

**Table 1:** Distribution of housing conditions in 559 Norwegian dairy herds by DD category (DD+/DD−) stratified on housing system

Housing	Free stalls		Tie stalls	
	DD+ (n = 108)	DD− (n = 281)	DD+ (n = 5)	DD − (n = 165)
	n (%)^b^	n (%)^b^	n (%)^b^	n (%)^b^
Herd size^a^				
11.7–30.3	8 (7.4)	69 (24.5)	2 (40.0)	146 (88.5)
30.4–39.8	18 (16.7)	60 (21.4)	2 (40.0)	14 (8.5)
39.9–46.9	24 (22.2)	54 (19.2)	1 (20)	3 (4.6)
47.0–56.4	25 (23.1)	53 (18.9)	0	2 (1.2)
56.5 or more	33 (30.6)	45 (16.0)	0	0
Missing	0	0	0	0
Alley-flooring				
Solid concrete	22 (20.4)	39 (13.9)		
Slatted concrete	52 (48.1)	170 (60.7)		
Solid rubber	20 (18.5)	34 (12.1)		
Slatted rubber	6 (5.6)	19 (6.8)		
Mixed concrete /rubber	8 (7.4)	17 (6.1)		
Other flooring	0	1 (0.4)		
Missing	0	1		
Bedding in cubicles/tie stalls				
Rubber mats	61 (57.0)	186 (66.4)	4 (80.0)	161 (98.2)
Mattresses	39 (36.5)	77 (27.5)	1 (20.0)	2 (1.2)
Rubber mats and mattresses	7 (6.5)	14 (5.0)	0	0
Other bedding	0	3 (1.1)	0	1 (0.6)
Missing	1	1	0	1
Milking system^a^				
AMS	96 (88.9)	222 (79.0)	1 (20.0)	0
Milking parlour	12 (11.1)	57 (20.3)	0	0
Pipeline	0	2 (0.7)	4 (80.0)	165 (100)
Missing	0	0	0	0
Milk yield Kg^a^				
4872.3–7863.5	20 (18.5)	57 (20.3)	3 (60.0)	98 (59.5)
7863.6–8403.9	13 (12.0)	65 (23.2)	0	36 (21.8)
8404–8912.5	29 (26.9)	49 (17.4)	1 (20.0)	22 (13.3)
8912.6–9534.1	24 (22.2)	54 (19.2)	1 (20.0)	5 (3.0)
> 9534.2	22 (20.4)	56 (19.9)	0	4 (2.4)
Missing	0	0	0	0

^a^Data from NDHRS

^b^Missing not included in %

**Table 2:** Distribution of management characteristics in 559 Norwegian dairy herds by DD category (DD+/DD−) stratified on housing system

Management	Free stalls		Tie stalls	
	DD+ (n = 108)	DD− (n = 281)	DD+ (n = 5)	DD− (n = 165)
	n (%)^b^	n (%)^b^	n (%)^b^	n (%)^b^
Number of staff				
1	3 (2.8)	22 (7.9)	1 (20.0)	21 (12.8)
2	36 (33.3)	114 (40.7)	1 (20.0)	76 (46.3)
3	36 (33.3)	79 (28.2)	1 (20.0)	39 (23.8)
≥ 4	33 (30.6)	65 (23.2)	2 (40.0)	28 (17.1)
Missing	0	1	0	1
Annual cleaning of barn				
No	35 (32.7)	49 (17.5)	1 (20.0)	2 (1.2)
Yes	70 (65.4)	231 (82.5)	4 (80.0)	161 (98.8)
Uncertain	2 (1.9)	0	0	0
Missing	1	1	0	2
Litter in cubicles/tie stalls				
No	13 (12.0)	35 (12.5)	0 (0.0)	9 (5.5)
Yes	95 (88.0)	246 (87.5)	5 (100)	153 (93.9)
Uncertain	0	0	0	1 (0.6)
Missing	0	0	0	2
Cleaning routines in alleys:				
By steps of cows				
No	92 (85.2)	205 (73.2)		
Yes	16 (14.8)	75 (26.8)		
Missing	0	1		
Automatic scraper				
No	59 (54.6)	183 (65.4)		
Yes	49 (45.4)	97 (34.6)		
Missing	0	1		
Automatic robot				
No	68 (63.0)	183 (65.4)		
Yes	40 (37.0)	97 (34.6)		
Missing	0	1		
Manuel scraping				
No	68 (63.0)	148 (52.9)		
Yes	40 (37.0)	132 (47.1)		
Missing	0	1		
Summer pasture				
No	7 (6.5)	16 (5.7)	0	7 (4.2)
Yes	101 (93.5)	265 (94.3)	5 (100)	158 (95.8)
Missing	0	0	0	0
Use bull instead of ai^a^				
No	84 (77.8)	237 (84.3)	5 (100)	152 (92.1)
Yes	24 (22.2)	44 (15.7)	0	13 (7.9)
Missing	0	0	0	0

^a^Data from NDHRS

^b^Missing not included in %

**Table 3:** Distribution of claw trimming routines in 559 Norwegian dairy herds by DD category (DD+/DD−) stratified on housing system

Trimming routines	Free stalls		Tie stalls	
	DD+ (n = 108)	DD− (n = 281)	DD+ (n = 5)	DD− (n = 165)
	n (%)^b^	n (%)^b^	n (%)^b^	n (%)^b^
Certified prof trimmer trims ≥ 90% of cows^a^				
No	5 (4.6)	42 (14.9)	0	24 (14.5)
Yes	103 (95.4)	239 (85.1)	5 (100)	141 (85.5)
Missing	0	0	0	0
Uncertified prof trimmer trims ≥ 30% of cows^a^				
No	104 (96.3)	259 (92.2)	5 (100)	150 (90.9)
Yes	4 (3.7)	22 (7.8)	0	15 (9.1)
Missing	0	0	0	0
Owner trims ≥ 30% of cows^a^				
No	108 (100.0)	260 (92.5)	5 (100)	156 (94.5)
Yes	0	21 (7.5)	0	9 (5.5)
Missing	0	0	0	0
Trimming frequency ^a^				
< 1/year	10 (9.3)	66 (23.5)	0	65 (39.4)
1<2/year	33 (30.6)	116 (41.3)	2 (40.0)	81 (49.1)
2<3/year	48 (44.4)	85 (29.2)	3 (60.0)	19 (11.5)
≥ 3/year	17 (15.7)	14 (5.0)	0	0
Missing	0	0	0	0
Cleaning of feet in chute before trimming				
No	54 (50.0)	179 (63.7)	2 (40.0)	93 (56.4)
Yes	47 (43.5)	69 (24.6)	3 (60.0)	53 (32.1)
Uncertain	7 (6.5)	33 (11.7)	0	19 (11.5)
Missing	0	0	0	0
All cows trimmed/ examined at routine trimming				
No	11 (10.2)	39 (13.9)	0	9 (5.6)
Yes	97 (89.8)	241 (86.1)	5 (100)	152 (94.4)
Missing	0	1	0	4
All pregnant heifers trimmed/ examined at routine trimming				
No	78 (72.9)	206 (74.1)	3 (60.0)	96 (58.9)
Yes	29 (27.1)	70 (25.2)	2 (40.0)	64 (39.3)
Uncertain	0	2 (0.7)	0	3 (1.8)
Missing	1	3	0	2
Trimming chute and equipment look clean at arrival				
No	1 (0.9)	1 (0.3)	0	1 (0.6)
Yes	104 (96.3)	275 (97.9)	5 (100)	161 (98.8)
Uncertain	3 (2.8)	5 (1.8)	0	1 (0.6)
Missing	0	0	0	2

^a^Data from NDHRS

^b^Missing not included in %

**Table 4:** Distribution of biosecurity characteristics in 559 Norwegian dairy herds by DD category (DD+/DD−) stratified on housing system

Biosecurity—contact with other herds	Free stalls		Tie stalls	
	DD+ (n = 108)	DD− (n = 281)	DD+ (n = 5)	DD− (n = 165)
	n (%)^b^	n (%)^b^	n (%)^b^	n (%)^b^
Staff working in other cattle herds				
No	59 (55.1)	150 (53.4)	3 (60.0)	72 (44.2)
Yes	46 (43.0)	130 (46.3)	2 (40.0)	91 (55.8)
Uncertain	2 (1.9)	1 (0.3)	0	0
Missing	1	0	0	2
Purchased cattle the last 5 years^a^				
0 animals	23 (21.3)	101 (35.9)	0	54 (32.7)
1–2 animals	12 (11.1)	31 (11.0)	2 (40.0)	32 (19.4)
3–4 animals	6 (5.6)	21 (7.5)	0	19 (11.5)
5–11 animals	19 (17.6)	49 (17.4)	2 (40.0)	34 (20.6)
12–27 animals	20 (18.5)	43 (15.3)	1 (20.0)	17 (10.3)
28–52 animals	15 (13.9)	23 (8.2)	0	7 (4.2)
≥53 animals	13 (12.0)	13 (4.6)	0	2 (1.2)
Missing	0	0	0	0
Require claw health documentation before purchase				
No	44 (55.7)	81 (48.8)	0	57 (56.4)
Yes	28 (35.4)	79 (47.6)	4 (100)	39 (38.6)
Uncertain	7 (8.9)	6 (3.6)	0 (	5 (5.0)
Missing	29	115	1	64
Claw health documentation required on				
Individual level	7 (25.0)	13 (16.9)	0	9 (23.1)
Herd level	11 (39.3)	27 (35.1)	1 (25.0)	10 (25.6)
Both individual cow and herd level	9 (32.1)	33 (42.8)	3 (75.0)	17 (43.6)
Uncertain	1 (3.6)	4 (5.2)	0	3 (7.7)
Missing	80	204	1	126
Purchased animals are in quarantine				
No	73 (91.2)	152 (92.7)	4 (100)	97 (96.0)
Yes	6 (7.5)	11 (6.7)	0	4 (4.0)
Uncertain	1 (1.3)	1 (0.6)	0	0
Missing	28	117	1	64
House animals from other herds				
No	103 (98.1)	276 (98.6)	5 (100)	159 (97.0)
Yes	2 (1.9)	4 (1.4)	0	5 (3.0)
Missing	3	1	0	1
Use shared pasture				
No	80 (79.2)	189 (71.3)	3 (60.0)	104 (65.8)
Yes	21 (20.8)	76 (28.7)	2 (40.0)	54 (34.2)
Missing	7	16	0	7
Shared transportation to pasture				
No	96 (96.0)	250 (94.3)	3 (60.0)	152 (96.2)
Yes	4 (4.0)	15 (5.7)	2 (40.0)	6 (3.8)
Missing	8	16	0	7
Biosecurity barrier in entrance of barn				
No	6 (5.6)	11 (3.9)	1 (20.0)	29 (17.6)
Yes	102 (94.4)	269 (95.7)	4 (80.0)	136 (82.4)
Uncertain	0	1 (0.4)	0	0
Missing	0	0	0	0
Facilities in biosecurity barrier:				
Hand wash in clean area				
No	31 (31.6)	66 (25.1)	1 (25.0)	45 (34.6)
Yes	67 (68.4)	197 (74.9)	3 (75.0)	85 (65.4)
Missing	10	18	1	35
Hand wash in unclean area				
No	26 (26.8)	92(35.7)	1 (33.3)	48 (37.5)
Yes	71 (73.2)	166 (64.3)	2 (67.7)	80 (62.5)
Missing	11	23	2	37
Clearly visible border between clean and unclean area				
No	29 (29.3)	71 (26.9)	2 (50.0)	30 (22.9)
Yes	70 (70.7)	193 (73.1)	2 (50.0)	101 (77.1)
Missing	9	17	1	34
Grid on each side of border between clean and unclean area				
No	70 (73.7)	186 (71.5)	3 (75.0)	99 (77.3)
Yes	25 (26.3)	74 (28.5)	1 (25.0)	29 (22.7)
Missing	13	21	1	37
Boots for visitors				
No	1 (1.0)	3 (1.1)	0	1 (0.7)
Yes	101 (99.0)	264 (98.9)	4 (100)	135 (99.3)
Missing	6	14	1	29
Coveralls for visitors				
No	1 (1.0)	2 (0.8)	0	2 (1.5)
Yes	101 (99.0)	263 (99.2)	4 (100)	133 (98.5)
Missing	6	16	1	30
Transport to slaughterhouse:				
The driver is never inside the barn	35 (32.4)	101 (35.9)	2 (40.0)	70 (42.4)
The driver is fetching the animals in restricted area inside the barn	23 (21.3)	55 (19.6)	2 (40.0)	9 (5.5)
Animals are fetched by the driver from the barn	24 (22.2)	43 (15.3)	0	62 (37.6)
The transport is parked partially inside the barn	6 (5.6)	20 (7.1)	1 (20.0)	4 (2.4)
Combination of the alternatives	8 (7.4)	21 (7.5)	0	6 (3.6)
None of the alternatives	12 (11.1)	41 (14.6)	0	14 (8.5)
Missing	0	0	0	0

^a^Data from NDHRS

^b^Missing not included in %

**Table 5:** Distribution of secondary control measures characteristics in 389 Norwegian dairy free-stall herds by DD category (DD+/DD−)

Secondary control measures	n	DD+ (n = 108)	DD− (n = 281)	OR	95% CI of OR	*P* value
		n (%)^b^	n (%)^b^			
Use of disinfectant litter in cubicles						
No	329	89 (82.4)	240 (85.4)	Baseline		0.464
Yes	60	19 (17.6)	41 (14.6)	1.25	0.69–2.27	
Missing	0	0	0			
Use of disinfectant footbaths						
No	354	88 (82.2)	266 (94.7)	Baseline		0.000
Yes	34	19 (17.8)	15 (5.3)	3.83	1.87–7.86	
Missing	1	1	0			
Installed claw washing system in barn						
No	321	78 (72.2)	243 (87.7)	Baseline		0.000
Yes	63	30 (27.8)	33 (11.9)	2.83	1.62–4.94	
Uncertain	1	0 (0)	1 (0.4)	1		
Missing	4	0	4			

*OR* odds ratio, *CI* confidence interval

^b^Missing not included in %

**Table 6:** Distribution of other infectious foot diseases in 389 Norwegian dairy free-stall herds by DD category (DD+ /DD−)

Other infectious claw diseases	n	DD+ (n = 108)	DD− (n = 281)	OR	95% CI of OR	*P* value
		n (%)^b^	n (%)^b^			
Heel horn erosion^a^						
0	151	20 (18.5)	131 (46.6)	Baseline		
1–10	142	39 (36.1)	103 (36.7)	2.48	1.36–4.51	0.003
11–30	58	28 (25.9)	30 (10.7)	6.11	3.04–12.28	0.000
> 30	38	21 (19.4)	17 (6.0)	8.09	3.66–17.90	0.000
Missing	0	0	0			
Interdigital dermatitis^a^						
0	179	18 (16.7)	161 (57.3)	Baseline		
1 –10	174	67 (62.0)	107 (38.1)	5.60	3.15–9.95	0.000
> 10	36	23 (21.3)	13 (4.6)	15.83	6.86–36.53	0.000
Missing	0	0	0			
Interdigital hyperplasia^a^						
0	295	59 (54.6)	236 (84.0)	Baseline		
1–5	82	40 (37.0)	42 (14.9)	3.81	2.27–6.40	0.000
> 5	12	9 (8.3)	3 (1.1)	12	3.15–45.71	0.000
Missing	0	0	0			
Interdigital phlegmon^a^						
0	356	92 (85.2)	264 (94.0)	Baseline		
1–3	30	14 (13.0)	16 (5.7)	2.51	1.18–5.35	0.017
4–6	3	2 (1.8)	1 (0.3)	5.74	0.51–64.04	0.156
Missing	0	0	0			

*OR *  odds ratio, *CI* confidence interval

^a^Data from NDHRS

^b^Missing not included in %

### Outcome variable and explanatory variables

The outcome variable was the herd’s DD status retrieved from the NDHRS. If one cow in the herd was recorded with a diagnosis of DD, the herd was classified as a case (DD+).

The explanatory variables were the answers in the questionnaire and data from NDHRS. When possible, open answers with comments were recoded into matching closed alternatives. For some questions with multiple answers, the number of categories was reduced by merging similar or few responses. For example, the answers “sand” and “straw” were merged with “others” in question “Describe the bedding in the cubicles/tie stalls” and the answers “4, 5 and 6 persons” were merged into one group “ ≥ 4 persons” in question “How many persons, including yourself, are working in the barn handling cattle?”.

Linearity between the outcome variable and all continuous variables was examined. If no linear association were seen transformation (e.g., log) of the explanatory variable was performed and linearity assessed again. Thereafter continuous variables were recoded into categorical variables. Cut-offs for continuous variables were based on either equally sized groups, percentiles, or previous experience. The number of cows in the herds was according to the free stalls grouped in five approximately equally sized groups as 11.7–30.3, 30.4–39.8, 39.9–46.9, 47.0–56.4, and  ≥ 56.5, and milk yield (kg) as 4872.3–7863.5, 7863.6–8403.9, 8404–8912.5, 8912.6–9534.1, and  > 9534.2 The average milk yield of all Norwegian dairy cows in 2019 was 8119 kg [32].

The continuous variable “Purchased cattle during the last 5 years” was categorized into seven groups: 0 animals, 1–2 animals, 3–4 animals, 5–11 animals, 12–27 animals, 28–52 animals, and  ≥ 53 animals. “Purchased cattle during the last 5 years” includes all registered animals purchased during the period Jan 2015 to Dec 2019, both dairy and beef, at all ages except calves. Calves were excluded based on assumed low risk for transferring DD between herds.

The variable “Trimming frequency” was created by dividing number of trimming events registered in NDHRS by the number of registered cows for each herd. One trimming event is equal to one recorded trimming and one cow can have  > 1 trimming event registered. The variables “Certified professional trimmer trims  ≥ 90% of the cows”, “Uncertified professional trimmer trims  ≥ 30% of the cows”, and “Owner trims  ≥ 30% of the cows” were created by dividing the number of registered trimming events by certified professional trimmer, uncertified professional trimmer, and owner respectively, by the total number of trimming events.

### Model building

Multilevel logistic regression was used to model the association between potential risk factors identified by literature studies and experienced causality, and variables related to the detection and diagnosis of DD, and the outcome. Due to few DD positive tie-stall herds, only the free-stall herds were included in the model. Questions with more than 15% missing answers/values were excluded from further analyses. Furthermore, the variables heel horn erosion, interdigital dermatitis, interdigital hyperplasia and interdigital phlegmon were regarded as competing outcome variables and not included in the analyses. Explanatory variables like “Use of disinfectant litter in cubicles/tie stalls”, “Use of disinfectant footbaths”, “Installed claw washing system in the barn”, and “Use of soap or disinfectant in claw washing system” are often not routinely used in Norwegian dairy herds, but frequently introduced as curative and preventive measures after a DD diagnose. Consequently, these secondary control measures were excluded from the multilevel logistic regression analyses. In total, 27 selected explanatory variables (out of 49), passed this initial screening steps.

Univariable associations were tested between each selected explanatory variable (one at a time) and the outcome variable (herd DD-status). This was performed using a univariable logistic regression model with a cut-off level for statistical significance of P < 0.2. The explanatory variables categorized as potential risk factors and variables related to the detection and diagnosis of DD were maintained for building the multilevel logistic regression model.

Twelve risk factor variables and variables related to the detection and diagnosis of DD with P < 0.2, were offered to the initial multilevel logistic regression model. Geographical area (county) was included in the model as a random effect. Possible correlation between explanatory variables (predictors) were assessed by visual inspection of scatter plots and box plots, but no correlations were found. A manual backwards elimination procedure was conducted to define the final model. In this procedure the variables with highest P-value were eliminated in a descending order until all remaining variables were significantly associated with the outcome (P < 0.05) or were considered as potential confounders. Variables considered to be potential confounders were tested by running the model with and without the variables in question while changes in estimates were monitored. The likelihood ratio test (LRT) was used to evaluate significance of categorical variables.

Biological plausible interactions between predictors retained in the final model (Table 7) were tested, but none of them were significant. The amount of unexplained variance pertaining to the county level was calculated using a fixed error term [33].

**Table 7:** Variables in the final model with a significant effect on a dairy free-stall herd’s digital dermatitis status (n=388^b^)

Variable	OR	95% CI of OR	*P* value
Purchase of livestock during the last 5 years ^a^			
0 animals	Baseline		
1–2 animals	1.53	0.61–3.80	0.364
3–4 animals	1.65	0.53–5.18	0.389
5–11 animals	1.71	0.77–3.78	0.185
12–27 animals	2.30	1.03–5.12	0.042
28–52 animals	4.34	1.71–11.04	0.002
≥ 53 animals	5.39	1.90–15.35	0.002
Missing			
Trimming frequency ^a^			
1 < 2/year	Baseline		
< 1/year	0.41	0.17–0.97	0.043
2 < 3/year	1.58	0.87–2.85	0.130
≥ 3/year	4.09	1.57–10.69	0.004
Certified prof trimmer trims ≥ 90% of cows^a^			
No	Baseline		
Yes	3.98	1.32–11.95	0.014
Cleaning of feet in chute before trimming			
No	Baseline		
Yes	1.98	1.12–3.51	0.019
Uncertain	0.82	0.31–2.14	0.678
Slatted flooring			
Yes	Baseline		
No	2.36	1.30–4.26	0.005
Uncertain	1.29	0.48–3.48	0.614
Random effect county (var):	0.568	0.142–2.273	

Variables with P < 0.05 in the final multilevel logistic regression model

*n* number of herds, *OR* odds ratio, *CI* confidence interval

^a^Data from NDHRS ^b^One herd had a missing value for slatted flooring

## Results

### Study population and geographic distribution

In total 685 questionnaires were completed giving a response rate of 36%. Of the 685 respondents 18.3% (126/685) respondents were excluded from further analyses due to missing claw trimmer (122) or housing system (4) recordings in NDHRS, resulting in a final study population of 559 herds. Among these herds 27.8% (108/389) of the free stalls versus 2.9% (5/170) of the tie stalls were DD positive. The distribution of replies to each question and data from NDHRS by DD category (DD+/DD−) stratified on free- and tie-stall herds is presented in Tables 1–4.

Twenty-six percent (145/559) of all respondents were in the county of Trøndelag, 11% (62/559) in Oppland, 11% (60/559) in Rogaland, 10% (58/559) in Hedmark, and the remaining 42% were from 13 other counties. Of the respondents with DD+ herds, 39% (44/113) were in Trøndelag, 20% (23/113) in Rogaland, and the remaining 41% were from 13 other counties. There were no DD+ respondents from Aust-Agder and Finnmark. Geographic distribution and DD status of the 559 respondents stratified on free- and tie-stall herds are shown in Additional file 2.

### Housing conditions and management characteristics

The average size of all herds included in this study was 50.0 cows (SD ± 17.7) in category DD+ and 34.2 cows (SD ± 17.6) in category DD−. The average herds size in the free-stall herds was 51.0 cows (SD ± 17.4) in category DD+ and 42.3 cows (SD ± 16.5) in category DD−, compared to 28.9 cows (SD ± 5.5) in category DD+ and 20.4 cows (SD ± 8.3) in category DD− in the tie-stall herds.

Of the DD+ herds, 96% (108/113) were housed in free stalls compared to 63% (281/446) of the DD− herds. In free stalls with category DD+ automatic milking systems were installed in 89% (96/108) versus 79% (222/281) in category DD−. The mean production of milk yield per cow per year in the free stalls was 8739 kg (SD ± 1001 kg) in category DD+ and 8652 kg (SD ± 1091 kg) in category DD−. The mean production of milk yield per cow per year in the tie stalls was 7822 kg (SD ± 525 kg) in category DD+ and 7675 kg (SD ± 68 kg) in category DD−. The distribution of herds by housing conditions and management characteristics by DD category (DD+/DD−) stratified on housing system is listed in Table 1 and 2.

### Claw trimming routines

In the free stalls with category DD+, 95% (103/108) of the herds used a certified professional trimmer for  ≥ 90% of the cows, 4% (4/108) used an uncertified professional trimmer for  ≥ 30% of the cows, and in no herds the owner trimmed  ≥ 30% of the cows. In free stalls with category DD−, 85% (239/281) of the herds used a certified professional trimmer for  ≥ 90% of the cows, 8% (22/281) used an uncertified professional trimmer for  ≥ 30% of the cows, and in 7% (21/281) the owner trimmed  ≥ 30% of the cows.

Trimming was more frequent in free- versus tie-stall herds. Twenty percent (76/389) of the free-stall herds were trimmed  < 1/year versus 38% (65/170) of the tie-stall herds while 42% (164/389) of the free-stall herds was trimmed  ≥ 2/year versus 13% (22/170) of the tie-stall herds. In the free stalls with category DD+, trimming 2–3 times per year was most frequent in 44% (48/108) of the herds, followed by 1–2 trimmings per year in 31% (33/108) of the herds. In free stalls with category DD−, trimming 1–2 times per year was most frequent in 41% (116/281) of the herds, followed by 2–3 trimmings per year in 30% (85/281) of the herds.

Cleaning of the feet in the chute before trimming, was performed in 44% (47/108) of the free-stall herds in category DD+ versus 25% (69/281) of the herds in category DD−. The distribution of trimming routines by DD category (DD+/DD−) stratified on housing system is in Table 3.

### Biosecurity

In DD+ free-stall herds, 79% (85/108) of the farmers had bought cattle during the last 5 years compared to 64% (180/281) of the farmers of DD− free-stall herds. Thirty-five percent (28/79) of the respondents in the DD+ category and 48% (79/166) in the DD− category required claw health documentation before the purchase in these herds. Only 8% (6/80) of the responding farmers with category DD+ and 7% (11/164) of the farmers with category DD− free-stall herds housed the purchased cattle in quarantine after arrival.

For all herds including both free- and tie-stall herds, high missing numbers were found for questions regarding requirement of claw health documentation before purchase of cattle (30/113) and (179/446), the type of claw health documentation required (81/113) and (330/446), and if the purchased cattle were placed in quarantine or not (29/113) and (181/446) for category DD+versus DD−. The distribution of biosecurity characteristics by DD category (DD+/DD−) stratified on housing system is in Table 4.

### Secondary control measures

In the free-stall herds there was a significant association between digital dermatitis and the use of disinfectant footbaths (baseline: no, OR = 3.83 (95% CI 1.87–7.86) for category yes), and installation of claw washing system in the barn (baseline: no, OR = 2.83 (95% CI 1.62–4.94) for category yes). The distribution of secondary control measures by DD category (DD+/DD−) in free-stall herds are listed in Table 5.

### Other infectious claw diseases

The distribution of infectious claw diseases by DD category (DD+/DD−) in free-stall herds are listed in Table 6. In the free-stall herds there was a significant association between digital dermatitis and heel horn erosion [baseline: 0, OR = 2.48 (95% CI 1.36–4.51) for category 1–10 cows, OR = 6.11 (95% CI 3.04–12.28) for 11 to 30 cows, and OR = 8.09 (95% CI 3.66–17.90) for  > 30 cows], interdigital dermatitis (baseline: 0, OR = 5.60 (95% CI 3.15–9.95) for category 1–10 cows, and OR = 15.83 (95% CI 6.86–36.53) for  > 10 cows, interdigital hyperplasia [baseline: 0, OR = 3.81 (95% CI 2.27–6.40) for category 1–5 cows, and OR = 12.0 (95% CI 3.15–45.71) for  > 5 cows] and interdigital phlegmon (baseline 0, OR = 2.51 (95% CI 1.18–5.35) for category 1–3 cows). Common for all four infectious claw diseases were increasing OR for DD with increasing numbers of affected cows.

### Variables associated with digital dermatitis in the multilevel logistic regression model

Results from the final model for the free-stall herd population are shown in Table 7. The free-stall herd’s DD status recorded in the NDHRS was associated with purchase of cattle during the last 5 years [baseline: 0 animals, OR = 2.30 (95% CI 1.03–5.12) for category 12 to 27 animals, OR = 4.34 (95% CI 1.71–11.04) for 28 to 52 animals, and OR = 5.39 (95% CI 1.90–15.35) for  ≥ 53 animals]. The DD status was also associated with trimming frequency [baseline: 1 < 2/year, OR = 0.41 (95% CI 0.17–0.97) for category < 1/year, and OR = 4.09 (95% CI 1.57–10.69) for ≥ 3/year], whether the claw trimming was done by a certified professional trimmer or not [baseline: ≤ 90% of the cows, OR = 3.98 (95% CI 1.32–11.95) for category ≥ 90% of the cows], cleaning of feet in the chute before trimming [baseline: no cleaning, OR = 1.98 (95% CI 1.12–3.51) for category cleaning], and alley flooring [baseline: slatted floor (OR = 2.36 (95% CI 1.30–4.26) for category solid floor]. The county random effect was significant, and the amount of unexplained variation at the county level was 15%.

## Discussion

The main purpose of this case–control study was to identify factors associated with DD in Norwegian dairy herds by analysing questionnaire responses from 559 farmers and data from the NDHRS. We found that 96% of the DD positive herds were housed in free stalls versus 4% in tie stalls. In the free-stall herds purchase of cattle during the last 5 years, increased claw trimming frequency, use of certified professional trimmer, cleaning of the feet in the chute, and solid versus slatted flooring in the alleys were associated with higher OR of DD. Based on experience and previous studies, we will discuss our results from different aspects to cover possible causalities.

Seventy percent of the producers had free-stall barns and out of these, 27.8% were DD positive. Of the 170 producers with tie-stall barns, only 2.9% were DD positive. This agrees with Cramer et al. [16] who showed that DD is more frequent in free stalls compared to tie stalls. The difference could be explained by major differences in management in the two housing systems. Hygiene and cleaning are probably easier to perform in a tie-stall compared to a free-stall barn, making the feet cleaner, dryer and therefore less vulnerable to contagious claw diseases. Another important factor is the within-herd spread of DD, which is reduced in a tie-stall barn where the animals are fixed to separate tie stalls for most of the year, compared to a free-stall barn where animals are mixed and walking freely around in the alleys and have direct contact with other cows with DD. Claw trimming is the most important tool for detection and diagnosis of DD and reduced trimming frequency in tie stalls may have resulted in false negative herds. In a Swedish study including both tie stalls and some free stalls Manske et al. [34] found no association between two versus one trimming per year and heel horn erosion and dermatitis. In the present study 80% the free-stall herds had a trimming frequency of  ≥ 1/year, compared to 62% in the tie-stall herds. Herd size is another important factor that differs between free stalls versus tie stalls. In Norway the average herd size is 40 cows in free-stall and 18 cows in tie-stall herds [35].

Purchase of cattle in the free-stall herds was associated with increased odds for DD. The risk for positive DD status increased significantly with purchase of 12–27 animals (OR = 2.30), 28 to 52 animals (OR = 4.34), and  ≥ 53 animals (OR = 5.39) during the last 5 years compared to no purchase. This agrees with several studies, which have shown that introduction of new animals is associated with higher odds of DD [18, 21]. Vanhoudt et al. [36] documented in a risk assessment study of DD that increasing number of purchased cattle was an important risk factor. Among the present open free-stall herds (purchase of ≥ 1 animal), only 6.4% of the producers performed quarantine procedures and 40.4% of the producers required claw health documentation before receiving new animals into their own herd. Bovine digital dermatitis treponemes can be transferred between farms directly with the DD lesions, but also by the animals’ oral cavity, gut, and rectum [37, 38]. In a Danish study, Klitgaard et al. [39] found DD associated *Treponema* spp. in slurry from herds with DD, and the disease may be transferred by purchased cows with manure on their skin. It is also documented that these treponemes can invade wounds like sole ulcers, hock, udder, and teat injuries [3, 40]. The importance of closed herds (no purchase of animals) is emphasized in the “5 Point Plan for Control of Digital Dermatitis” [41]. This systematic plan to control DD elucidates that poor external biosecurity routines are important risk factors.

Claw trimming frequency in the free-stall herds was associated with DD, trimming  < 1 time per year (OR = 0.41) and trimming  ≥ 3 times per year (OR = 4.09). The relationship between increased trimming frequency and DD might imply a risk for spreading the disease [20]. At the same time, more frequent trimming means a more frequent inspection of individual animals and increased opportunity to detect DD lesions [11]. Frequent inspection of the feet is important because DD has a dynamic clinical appearance that varies over the course of the disease, making detection and diagnostics challenging [42]. On the other hand, absence or long trimming intervals might also in contrast to the present study result in higher DD prevalence due to inadequate topical curative treatment which is recommended to control the disease [5, 43].

Use of a certified professional trimmer for more than 90% of the cows versus not in the present free-stall herds was associated with higher odds for a positive DD status (OR = 3.98). The reason for higher odds ratio when using a certified professional trimmer may be that trimmers visiting a lot of herds could be a potential risk factor for introducing DD to naïve herds. During claw trimming session, the chute, grinder, hoof knife, and hoof tester are in close contact with DD lesions and manure, making them potential vectors for transmission of *Treponema* spp. if not washed and disinfected properly between different herds. Sullivan et al. [20] found that treponemes from one or more of the three phylogroups associated with DD were present in at least 42% of swabs from knives used to trim the claws of cattle with DD lesions, and another study on contagious ovine digital dermatitis has shown that DD associated *Treponema* spp. can survive on rubber gloves for 3 days [44]. Another important reason may be that Norwegian certified trimmers are well-educated making their DD registration more correct versus other trimmers. Certified Norwegian claw trimmers are required to record all their findings during trimming (app or paper), and this does not apply for other trimmers nor producers trimming their own cows. In addition, producers and other trimmers may have inadequate knowledge about DD which also may result in limited recorded DD cases in NDHRS. One could therefore argue, that certified professional trimmers probably both are valuable resources for detection of DD and important risk factors for transferring the disease to naïve herds if routines for washing and disinfection are applied inadequately.

Free-stall herds where feet were cleaned before trimming had higher odds for DD compared to free-stall herds where no cleaning was performed (OR = 1.98). The most probable reason for this is that cleaning makes it easier to detect small DD lesions compared to when the feet are covered with manure. To characterize the different DD lesions, a M-stage scoring system is developed, where clinical stages of DD are scored based on size and whether the lesions are active, chronic, or healed [1]. Of 937 trimmed cows in 21 Norwegian dairy herds Ahlén & Fjeldaas [28] found that 57.6% were DD negative, 22.1% had M1 lesions, 2.9% had M2 lesions, 3.6% had M3 lesions, 3.8% had M4 lesions and 10% had M4.1 lesions. They also found that many small M1 lesions were in the interdigital cleft. In their study all hind feet were cleaned with running water or wet napkins making visualisation and diagnostics significantly easier. Small DD lesions like M1 are difficult to detect, and when located in the interdigital cleft, basically impossible to see without cleaning and proper lighting [28, 42]. Stokes et al. [45] found a very high sensitivity (1.0) and specificity (0.99) when diagnosing DD with a borescope after cleaning the feet with a hose and drying them with a paper towel. Farmers of herds recorded with DD, probably have more focus on the importance of washing the feet before trimming, which also may contribute to the association between DD and cleaning of the feet in the present study. Another hypothesis for higher odds of DD related to cleaning of the feet before trimming, is that washing equipment is a risk factor, transferring infection between individual cows within a herd and between herds. However, this probability is limited because Norwegian claw trimmers usually use a water hose from the barn, rather than their own washing equipment. Consequently, cleaning of the feet before trimming seems to be an effective tool for detection of the disease, rather than a risk factor.

In the free-stall herds, solid versus slatted flooring in the alleys was associated with higher odds for a positive DD status (OR = 2.36). This finding partly agrees with a previous Norwegian study by Fjeldaas et al. [46] who found fewer cases of dermatitis including both interdigital and digital dermatitis on slatted versus solid flooring. Our finding also agrees with a Dutch study by Somers et al. [47] who found that cows on slatted floor had significantly less DD than cows on solid concrete floor and grooved floor. In another study, Somers et al. [43] also recorded less DD in herds with slatted floors with scrapers in the alleys compared with DD in herds with solid floor with scrapers. The reason is probably more urine and manure in alleys with solid versus slatted flooring, which results in increased growth and transmission of bacteria.

In the present study, considering factors associated with DD in Norwegian dairy herds we chose the professional trimmers recordings in NDHRS for assessment of the DD status. Their NDHRS recordings are based on scored lesions on the cows’ feet at routine trimming in a chute, and are the “gold standard” for clinical diagnosis of DD. This is supported by ICARs global standard for livestock data, Section 7—Guidelines for health, female fertility, udder health, claw health traits and lameness in bovine [3]. However, a possible bias is that the trimmers do not recognize small DD lesions, especially when the feet are covered by manure, or the lesions are hidden in the interdigital cleft. Overestimation of DD lesions is in our opinion most unlikely. In a most-recent Norwegian study the sensitivity and specificity of recording DD at trimming in a chute was estimated to 0.76–0.80 and 0.87–0.88, respectively [48]. Most Norwegian claw trimmers undergo a certification course including theory classes, field work with guided mass training, and an exam before graduating and receiving a certificate. Their diagnoses are guided by the Nordic claw health atlas [31].

Even though Norwegian legislation require that all dairy cows are housed in free stalls by 2034 [30], we chose to have no exclusion criteria when selecting our study population. In 2020, 35% of the Norwegian dairy cows were still housed in tie stalls, and by including all dairy herds we had the opportunity to compare DD in free stalls versus tie stalls on national level.

The questionnaire was sent to the farmer/owner of the herds. In Norway with relatively small dairy herds, the farmers usually know their herds and individual cows well and are competent to answer the questions correctly. No difference in ratio between DD positive and DD negative herds in the total population receiving the questionnaire, 380/1530 = 0.25 versus 113/446 = 0.25 among the respondents, indicates no bias regarding who answered the questions even though one could suspect that farmers with DD+ herds might be more interested in answering the questionnaire versus those with DD− herds. Stratified random sampling of controls on housing system could probably have recruited more DD negative free-stall herds.

A possible weakness of the present study is the inclusion of all three categories of claw trimmers: certified professional trimmers, uncertified professional trimmers, and farmers. One could argue that only herds trimmed by certified professional trimmers should have been included, because they are better educated and trained in detection and registration of DD. On the other hand, it was interesting to analyse if they registered differently compared to the other two categories. As described above, uncertified professional trimmers and farmers are not required to register claw diseases, which may have underestimated the actual DD status in the study population.

## Conclusions

Digital dermatitis was far more frequent in Norwegian dairy herds housed in free stalls versus those housed in tie stalls. Factors associated with increased odds of digital dermatitis within free-stall herds were purchase of cattle, increasing claw trimming frequency, use of certified professional trimmer, cleaning of the feet in the chute before trimming, and solid versus slatted flooring in the alleys.

## Supplementary Information

**Additional file 1. **English version of farmer questionnaire in Questback used to obtain data in this Norwegian case-control study of bovine digital dermatitis.

**Additional file 2. **Geographic distribution of respondents (n) by DD category (DD+/DD−) for each county in two separate diagrams, on free-stall herds (n=389) and tie-stall herds (n=170). N=559.

## Data Availability

The datasets used and/or analyzed during the current study are available from the corresponding author on reasonable request.

## Abbreviations

DD: Digital dermatitis
NDHRS: Norwegian dairy herd recording system
