# Supplementary material for: A case–control study regarding factors associated with digital dermatitis in Norwegian dairy herds
Source: Acta Vet Scand. 2022 Aug 13;64:19. doi: 10.1186/s13028-022-00635-0 (PMC9375421; doi:10.1186/s13028-022-00635-0)
Supplement: Supplementary file 1 — Additional file 1. English version of farmer questionnaire in Questback used to obtain data in this Norwegian case-control study of bovine digital dermatitis. [file 13028_2022_635_MOESM1_ESM.pdf]

Additional file 1: Farmer questionnaire used to obtain data of bovine digital dermatitis in Norwegian dairy herds

Information about the herd:

Name of farmer: ……

Herd identity (8 numbers):...

The usefulness of this study will be improved if you provide your farmer identity. All responses and results will be handled and presented confidentially.

Management:

1. How many persons, including yourself, are working in the barn handling the cattle? Choose one alternative.
   ☐ 1
   ☐ 2
   ☐ 3
   ☐ 4
   ☐ 5
   ☐ 6 or more

2. Do any of these also work with other cattle herds? Choose one alternative.
   ☐ Yes
   ☐ No
   ☐ I don't know

3. Which type of alley-flooring dominates in your barn? Choose one alternative.
   ☐ Solid concrete
   ☐ Slatted concrete
   ☐ Solid rubber
   ☐ Slatted rubber
   ☐ A mix of concrete and rubber
   ☐ Others

4. Describe the cleaning routines of the alleys in your barn. Choose one or more alternatives.
   ☐ By the steps of the cows
   ☐ Automatic manure scraper
   ☐ Automatic manure robot
   ☐ Manual scraping
   ☐ Truck/tractor
   ☐ Other (please specify):
   …..……………………………………………………………………………………….

5. Describe the bedding in the cubicles / tie stalls. Choose one or more alternatives.
   ☐ Rubber mats
   ☐ Matresses
   ☐ Sand
   ☐ Concrete
   ☐ Other (please specify):
   ……………………………………………………………………………………………

6. Do you use litter in your cubicles / tie stalls? Choose one alternative.
   ☐ Yes
   ☐ No
   ☐ I don't know

7. Do you use disinfectant litter in your cubicles / tie stalls? Choose one alternative.
   ☐ Yes
   ☐ No

8. Have you installed an automatic milking system (AMS)? Choose one alternative.
   ☐ Yes
   ☐ No

9. Have you installed a claw washing system in your AMS or at other locations in your barn? Choose one alternative.
   ☐ Yes
   ☐ No *(move on to question 11)*
   ☐ I don't know *(move on to question 11)*

10. If yes, do you use soap or disinfection in your claw washing system? Choose one alternative.
    ☐ Yes
    ☐ No, only water
    ☐ Others

11. Do you use disinfectant footbaths for your dairy cows? Choose one alternative.
    ☐ Yes
    ☐ No *(move on to question 14)*
    ☐ I don't know *(move on to question 14)*

12. How long have you been using disinfectant footbaths? Choose one alternative.
    - ☐ Less than 1 month
    - ☐ 1-6 months
    - ☐ 7-12 months
    - ☐ More than 1 year

13. How often do your cows walk through the footbath? Choose one alternative.
    - ☐ Twice daily
    - ☐ Once daily
    - ☐ Several times a week
    - ☐ Once a week
    - ☐ Several times a month
    - ☐ Once a month
    - ☐ Once every second week
    - ☐ Once every 6. month
    - ☐ Other (please specify):

      …………………………………………………………………………………………………

14. Do you perform annual cleaning of the barn? Choose one alternative.
    - ☐ Yes
    - ☐ No
    - ☐ I don't know

Biosecurity – contact with other cattle herds

15. Have you purchased cattle during the last 5 years? Choose one alternative.
    - ☐ Yes
    - ☐ No *(move on to question 21)*
    - ☐ I don't know (*move on to question 21)*

16. If yes, when did the last animal arrive? Choose one alternative.
    - ☐ During the last year
    - ☐ During the last 2 years
    - ☐ During the last 3 years
    - ☐ During the last 4 years
    - ☐ During the last 5 years
    - ☐ I don't know

17. Are these cattle bought from permanent suppliers? Choose one alternative.
    - ☐ Yes
    - ☐ No
    - ☐ I don't know

18. Do you require claw health documentation before purchase of livestock? Choose one alternative.
    - ☐ Yes
    - ☐ No *(move on to question 20)*
    - ☐ I don't know *(move on to question 20)*

19. If yes, what kind of claw health documentation do you require before purchase of new animals? Choose one alternative.
    - ☐ On individual cow level
    - ☐ On herd level
    - ☐ On both individual cow and herd level
    - ☐ I don't know

20. Are purchased animals placed in quarantine before they enter your herd? Choose one alternative.
    - ☐ Yes
    - ☐ No
    - ☐ I don't know

21. Do you sometimes house animals from other herds, for example to fill your milk quota? Choose one alternative.
    - ☐ Yes
    - ☐ No
    - ☐ I don't know

22. Are all cattle or parts of your herd on summer pasture? Choose one alternative.
    - ☐ Yes
    - ☐ No *(move on to question 25)*
    - ☐ I don't know *(move on to question 25)*

23. Does your herd share pasture with other herds? Choose one alternative.
    - ☐ Yes
    - ☐ No
    - ☐ I don't know

24. Does your herd share transport to pasture with other herds? Choose one alternative.
    - ☐ Yes
    - ☐ No
    - ☐ I don't know

25. Transport to the slaughterhouse. Which alternatives are correct for your herd? Choose one or more alternatives.
    - ☐ The slaughterhouse-transport driver is never inside my barn and I am never entering the transport
    - ☐ The slaughterhouse-transport driver is fetching the animals in a restricted area inside my barn
    - ☐ Animals are fetched by the slaughterhouse-transport driver from the barn
    - ☐ The transport is parked partially inside the barn
    - ☐ None of the alternatives (please specify):
      ………………………………………………………………………………………………

Claw trimming routines and claw health

26. On average, how many times are your cows trimmed each year? Choose one alternative.
    - ☐ Less than once
    - ☐ Once
    - ☐ Twice
    - ☐ Three times
    - ☐ Four times
    - ☐ Other alternatives (please specify):
      ……………………………………………………………………………………………..

27. Who performs the trimming? Choose one or more alternatives.
    - ☐ Certified professional trimmer
    - ☐ Uncertified professional trimmer
    - ☐ The farmer or members of the staff
    - ☐ Others (please specify):
      ………………………………………………………………………………………………

28. Are all dairy cows trimmed / examined in the chute at each routine trimming? Choose one alternative.
    - ☐ Yes
    - ☐ No
    - ☐ I don’t know

29. Are all pregnant heifers trimmed / examined in the chute at each routine trimming? Choose one alternative.
    - ☐ Yes
    - ☐ No
    - ☐ I don’ know

30. Are the cows’ feet cleaned in the chute before trimming? Choose one alternative.
    ☐ Yes
    ☐ No *(move on to question 32)*
    ☐ I don’ know *(move on to question 32)*

31. If yes, how many of the trimmed animals get their feet cleaned? Choose one alternative.
    ☐ All
    ☐ Most
    ☐ Approximately half of them
    ☐ Less than half of them
    ☐ Only a few
    ☐ I don’t know

32. Do any of your cows have the foot disease digital dermatitis? Choose one alternative.
    ☐ Yes
    ☐ No
    ☐ I don’t know

33. Do the trimming chute and trimming equipment look clean when the professional trimmer arrives? Choose one alternative.
    ☐ Yes
    ☐ No
    ☐ I don’t know

Biosecurity barrier

34. Is there a biosecurity barrier in the entrance of your barn? Choose one alternative.
    ☐ Yes
    ☐ No *(you have finished the questionnaire)*
    ☐ I don’t know *(you have finished the questionnaire)*

35. If yes, mark the facilities installed:

Type of facility	***Yes***	***No***
Handwash with soap in clean area		
Handwash with soap in unclean area		
Visible and elevated border between clean and unclean area		
Grid on each side of border separating clean and unclean area		
Boots for visitors		
Coveralls for visitors		

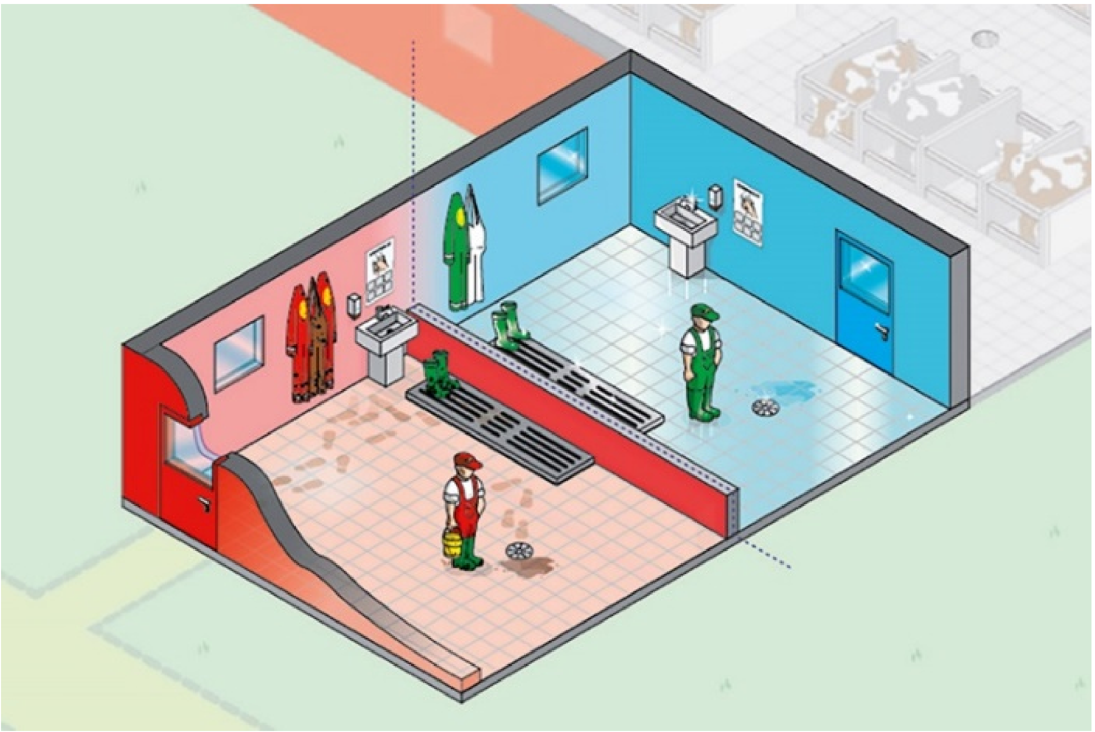

Photo from Animalia AS and Koorimp's pamphlet "Biosecure".
