# Supplementary material for: A case–control study regarding factors associated with digital dermatitis in Norwegian dairy herds
Source: Acta Vet Scand. 2022 Aug 13;64:19. doi: 10.1186/s13028-022-00635-0 (PMC9375421; doi:10.1186/s13028-022-00635-0)
Supplement: Supplementary file 2 — Additional file 2. Geographic distribution of respondents (n) by DD category (DD+/DD−) for each county in two separate diagrams, on free-stall herds (n=389) and tie-stall herds (n=170). N=559. [file 13028_2022_635_MOESM2_ESM.pdf]

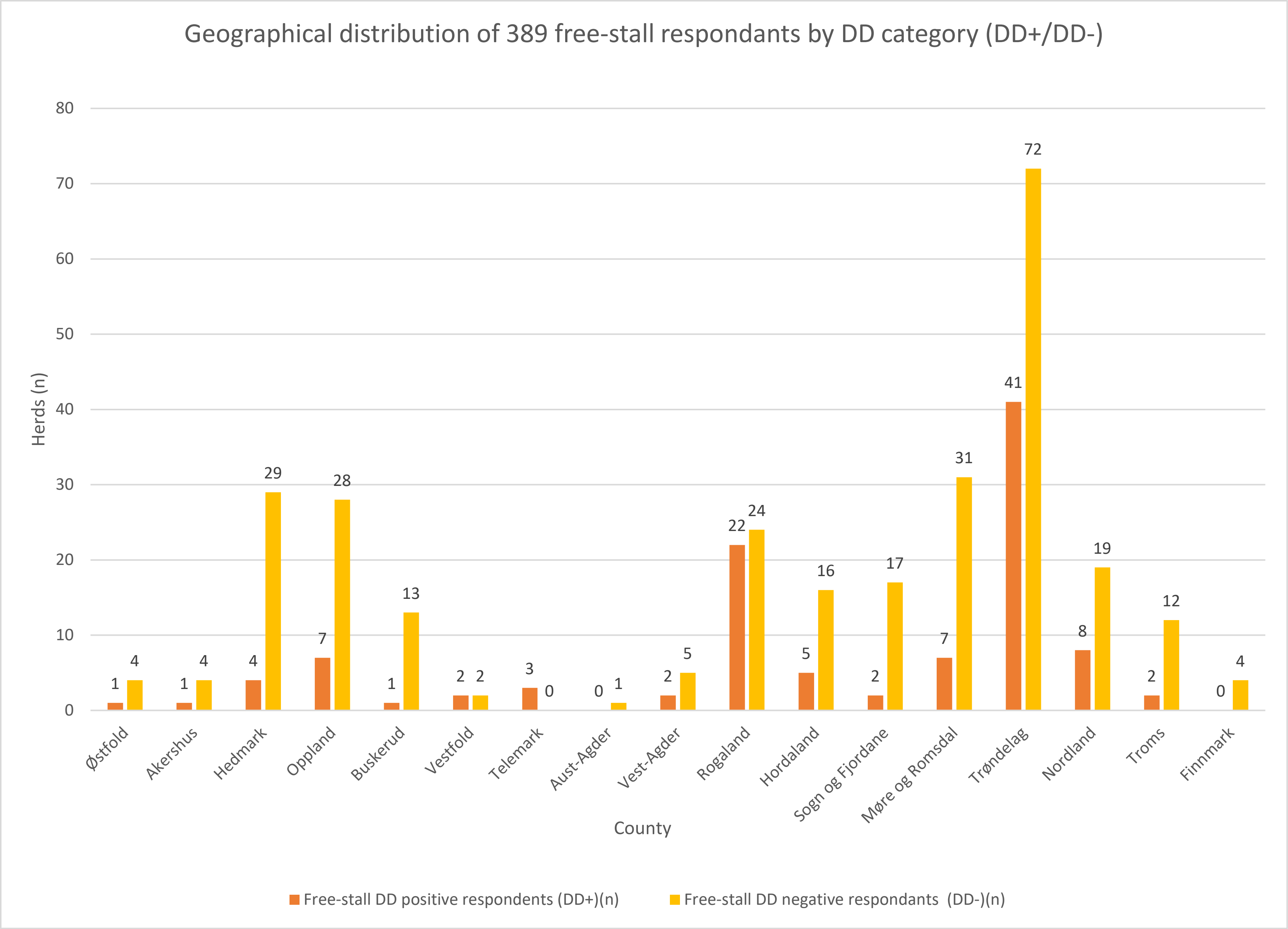

Geographical distribution of 389 free-stall respondants by DD category (DD+/DD-)
Herds (n)
0
10
20
30
40
50
60
70
80
1
4
1
4
4
29
7
28
1
13
2
2
3
0
0
1
2
5
22
24
5
16
2
17
7
31
41
72
8
19
2
12
0
4
Østfold
Akershus
Hedmark
Oppland
Buskerud
Vestfold
Telemark
Aust-Agder
Vest-Agder
Rogaland
Hordaland
Sogn og Fjordane
Møre og Romsdal
Trøndelag
Nordland
Troms
Finnmark
County
Free-stall DD positive respondents (DD+)(n)
Free-stall DD negative respondants (DD-)(n)

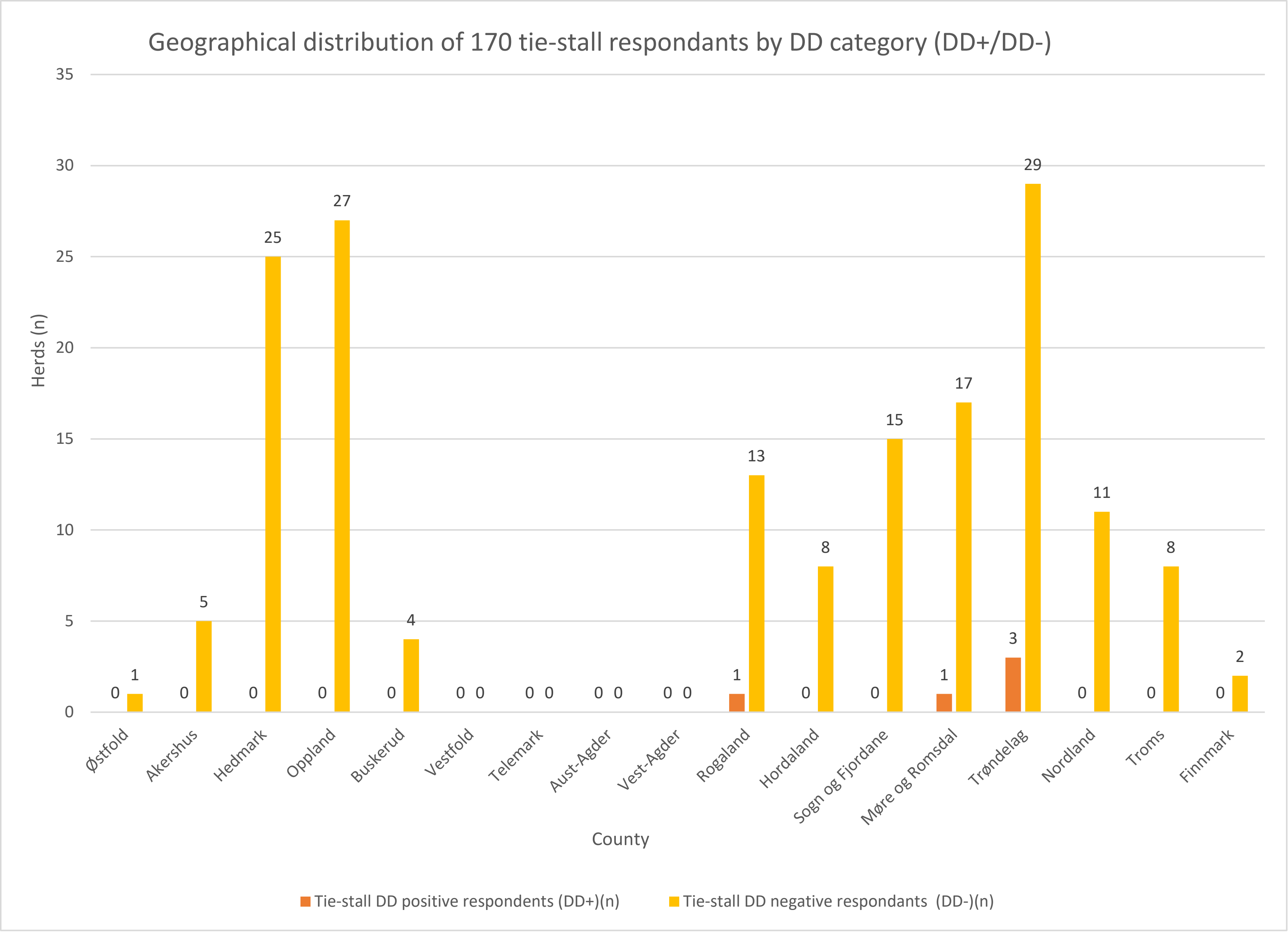

Geographical distribution of 170 tie-stall respondants by DD category (DD+/DD-)
Herds (n)
35
30
25
20
15
10
5
0
0 1
0 5
0 25
0 27
0 4
0 0
0 0
0 0
0 0
1 13
0 8
0 15
1 17
3 29
0 11
0 8
0 2
Østfold
Akershus
Hedmark
Oppland
Buskerud
Vestfold
Telemark
Aust-Agder
Vest-Agder
Rogaland
Hordaland
Sogn og Fjordane
Møre og Romsdal
Trøndelag
Nordland
Troms
Finnmark
County
Tie-stall DD positive respondents (DD+)(n)
Tie-stall DD negative respondants (DD-)(n)

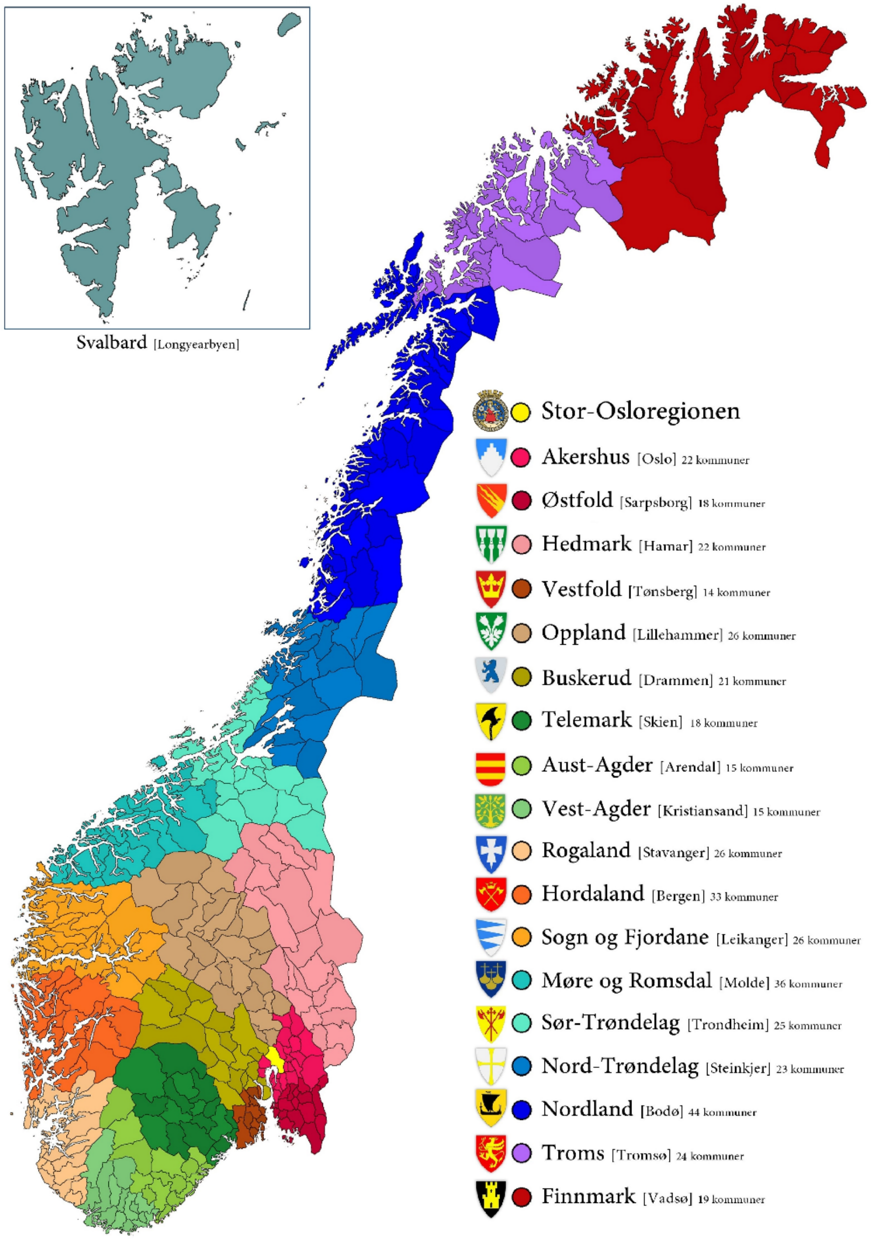
Svalbard [Longyearbyen]
Stor-Osloregionen
Akershus [Oslo] 22 kommuner
Østfold [Sarpsborg] 18 kommuner
Hedmark [Hamar] 22 kommuner
Vestfold [Tønsberg] 14 kommuner
Oppland [Lillehammer] 26 kommuner
Buskerud [Drammen] 21 kommuner
Telemark [Skien] 18 kommuner
Aust-Agder [Arendal] 15 kommuner
Vest-Agder [Kristiansand] 15 kommuner
Rogaland [Stavanger] 26 kommuner
Hordaland [Bergen] 33 kommuner
Sogn og Fjordane [Leikanger] 26 kommuner
Møre og Romsdal [Molde] 36 kommuner
Sør-Trøndelag [Trondheim] 25 kommuner
Nord-Trøndelag [Steinkjer] 23 kommuner
Nordland [Bodø] 44 kommuner
Troms [Tromsø] 24 kommuner
Finnmark [Vadsø] 19 kommuner
